# Genetic Dissection of the Regulatory Mechanisms of *Ace2* in the Infected Mouse Lung

**DOI:** 10.3389/fimmu.2020.607314

**Published:** 2021-01-08

**Authors:** Fuyi Xu, Jun Gao, Silke Bergmann, Amy C. Sims, David G. Ashbrook, Ralph S. Baric, Yan Cui, Colleen B. Jonsson, Kui Li, Robert W. Williams, Klaus Schughart, Lu Lu

**Affiliations:** ^1^ Department of Genetics, Genomics, and Informatics, University of Tennessee Health Science Center, Memphis, TN, United States; ^2^ Institute of Animal Husbandry and Veterinary Science, Shanghai Academy of Agricultural Sciences, Shanghai, China; ^3^ Department of Microbiology, Immunology, and Biochemistry, University of Tennessee Health Science Center, Memphis, TN, United States; ^4^ Department of Epidemiology, University of North Carolina at Chapel Hill, Chapel Hill, NC, United States; ^5^ Department of Microbiology and Immunology, University of North Carolina at Chapel Hill, Chapel Hill, NC, United States; ^6^ Department of Infection Genetics, Helmholtz Centre for Infection Research, Braunschweig, Germany; ^7^ University of Veterinary Medicine Hannover, Hannover, Germany

**Keywords:** H1N1, acute lung injury, BXD family, *Ace2*, host response, viremia network

## Abstract

Acute lung injury (ALI) is an important cause of morbidity and mortality after viral infections, including influenza A virus H1N1, SARS-CoV, MERS-CoV, and SARS-CoV-2. The angiotensin I converting enzyme 2 (ACE2) is a key host membrane-bound protein that modulates ALI induced by viral infection, pulmonary acid aspiration, and sepsis. However, the contributions of *ACE2* sequence variants to individual differences in disease risk and severity after viral infection are not understood. In this study, we quantified H1N1 influenza-infected lung transcriptomes across a family of 41 BXD recombinant inbred strains of mice and both parents—C57BL/6J and DBA/2J. In response to infection *Ace2* mRNA levels decreased significantly for both parental strains and the expression levels was associated with disease severity (body weight loss) and viral load (expression levels of viral NA segment) across the BXD family members. Pulmonary RNA-seq for 43 lines was analyzed using weighted gene co-expression network analysis (WGCNA) and Bayesian network approaches. *Ace2* not only participated in virus-induced ALI by interacting with TNF, MAPK, and NOTCH signaling pathways, but was also linked with high confidence to gene products that have important functions in the pulmonary epithelium, including *Rnf128*, *Muc5b*, and *Tmprss2*. Comparable sets of transcripts were also highlighted in parallel studies of human SARS-CoV-infected primary human airway epithelial cells. Using conventional mapping methods, we determined that weight loss at two and three days after viral infection maps to chromosome X—the location of *Ace2*. This finding motivated the hierarchical Bayesian network analysis, which defined molecular endophenotypes of lung infection linked to *Ace2* expression and to a key disease outcome. Core members of this Bayesian network include *Ace2, Atf4*, *Csf2*, *Cxcl2*, *Lif, Maml3*, *Muc5b*, *Reg3g*, *Ripk3*, and *Traf3*. Collectively, these findings define a causally-rooted *Ace2* modulatory network relevant to host response to viral infection and identify potential therapeutic targets for virus-induced respiratory diseases, including those caused by influenza and coronaviruses.

## Introduction

Five major influenza A virus (IAV) pandemics have swept through human populations in the last 130 years—the 1890 H3N8 pandemic, the devastating 1918 H1N1 pandemic (1), and serious global outbreaks in 1957 (H2N2), 1968 (H3N2), and the 2009 H1N1 swine influenza. The current COVID-19 pandemic is caused by a novel coronavirus, termed severe acute respiratory syndrome (SARS) coronavirus 2 (SARS-CoV-2). This pathogen has spread with unprecedented speed and efficiency from an initial outbreak in November or December 2019.

The primary pathological features of the influenza A outbreaks and both the 2002 SARS and current COVID-19 outbreaks are viral pneumonia and acute lung injury (ALI) (2). The well documented symptoms of influenza A (H1N1) and COVID-19 have substantial overlap. For both viral pathogens, infected humans can have symptoms ranging from mild coughing, congestion, and fever, to severe and lethal pulmonary edema and disseminating diseases of other organs. Individual host genetic differences almost certainly play an important role in the development and severity of the illness and its time course (3–7).

The human angiotensin I converting enzyme 2 (ACE2) is a cell membrane carboxypeptidase and negative modulator of the renin angiotensin system (RAS). This protein also has a key role in ciliary function in epithelial cells and is the principal receptor bound by spike proteins of SARS-CoV (8, 9) and SARS-CoV-2 (10). *ACE2* is highly expressed in nasal cavity epithelial cells, especially on apical cilia of small airway cells and in alveolar epithelial type II cells (AT2) (11, 12).

Paradoxically, while serving as a key node in cellular entry of SARS-CoV and SARS-CoV-2 virions, ACE2 also protects against lung disease in animal models (13). For example, treatment with recombinant human ACE2 protects against acute lung failure induced by acid aspiration or sepsis in *Ace2* knockout mice as well as in wild-type controls (14). Of note, *Ace2* expression in mouse lungs is downregulated following SARS-CoV infection. The spike protein alone induces ACE2 downregulation in cell culture and enhances the severity of ALI induced by acid aspiration in wildtype mice (8). In contrast, viral spike protein is unable to affect the severity of lung failure in *Ace2* knockout animals, indicating the effect of spike protein on acid-induced ALI is ACE2 specific (8). Interestingly, expression of ACE2 is also significantly downregulated by IAVs in infected cell cultures, although in this case, ACE2 is dispensable for viral entry and replication (15). In mice, *Ace2* deficiency is associated with aggravated ALI induced by IAV H7N9 or respiratory syncytial virus (16, 17). The precise mechanism underlying these findings is unclear, as is how alterations in ACE2 expression modulate the severity of pulmonary disease inflicted by coronaviruses and other IAV subtypes.

Expression of *ACE2* in human lung has an impressive level of variation among healthy humans. In the GTEx RNA-seq transcriptome survey (18), the maximum range of expression in the lung is about 22-fold, the interquartile range is 1.8-fold, and the coefficient of variation (standard deviation/mean) is high—nearly 0.6 (genenetwork.org/show_trait?trait_id=ENSG00000130234 &dataset=GTEXv8_Lung_tpm_0220). Given this finding, it is possible, even likely, that there are marked genetic differences in host susceptibility that are a function of numbers or ratios of ACE2-positive AT2 cells and epithelial cells in lung (11) or the level expression by these cells of *ACE2*. However, we emphasize that causal linkage between genetic variation in *ACE2* and to susceptibility or progress of infection is still poorly defined in humans, or even in mouse models.

Susceptibility to IAV, such as H1N1 and H7N7 influenza infections, varies greatly among strains of mice (19), and especially between the two key inbred strains we use here—C57BL/6J (B6), the reference strain for most genetic and molecular studies—and DBA/2J (D2), the oldest inbred strain of mice (20). B6 is relatively resistant to IAV and often survives infection. In contrast, D2 loses body weight rapidly, and at the same inoculum dose as given to B6 dies within 5–7 days. Our laboratory has generated and maintains approximately 120 BXD recombinant inbred (RI) strains derived from crosses between B6 and D2 parents. Collectively, this family of RI strains segregates for ~6 million variants with minor allele frequencies above 0.3 (21), making it an ideal reference population to model genetic variation in susceptibility to IAV infections (22). Indeed, in previous genetic mapping using the BXD family we have defined loci and even cloned gene variants that control the host response to IAV (H1N1) infection (23) and to other infectious diseases (24–27). Here, we have analyzed the lung transcriptome of 41 members of the family and both parental strains after infection with IAV (H1N1). We define patterns of covariation of RNA-seq data with induced variation in *Ace2* expression, as well as with outcome measures and with key molecular networks and endophenotypes likely to contribute to host defense and pathologies of H1N1 and possibly to SARS-CoV-induced ALI.

## Materials and Methods

### Mice

We used females from 41 BXD RI strains and both parental strains—B6 and D2. Mice were between 8 and 12 weeks of age when infected. They were housed and maintained on a 12:12 light/dark cycle, with *ad libitum* access to food and water.

### Virus

Original stocks of mouse-adapted A/Puerto Rico/8/34 (H1N1, PR8M) virus were obtained from Stefan Ludwig, University of Münster  (28). Virus stocks were propagated in the chorioallantoic cavity of 10-day-old pathogen-free embryonated chicken eggs for 48 h at 37°C as described previously (29). Viral titer was determined using a focus-forming unit (FFU) assay as described previously (29).

### Infection of Mice

Animals were anesthetized by intraperitoneal injection of ketamine/xylazine (10 % (v/v) of 100 mg/ml ketamine and 5 % (v/v) of 20 mg/ml xylazine in 0.9 % (w/v) NaCl with a dose adjusted to body weight (200 µl/20 g body weight). Infection was performed by intranasal application of virus solution in 20 µl sterile phosphate-buffered saline (PBS), with a PR8M dosage of 2×10^3^ FFU. Mice were bred and infected at the animal facilities at UTHSC.

### RNA Isolation and Sequencing

Mice were sacrificed 3 days post-infection (dpi) and both lungs were extracted and transferred immediately to RNAlater (Qiagen), stored at 4°C for one day, and then stored at −20°C. RNA was isolated using Qiagen Midi kit (30). RNA quality was evaluated on a 2100 Bioanalyzer (Agilent). Five-hundred nanograms of total RNA was used to prepare libraries for sequencing using the Lexogen SENSE RNA-seq library kit for Ion Torrent. Libraries were amplified for 11 cycles as the final step of library preparation. Before sequencing, 1-µl aliquots were pooled and sequenced on an Ion Torrent PGM 314 chip. Barcoded data from the PGM was used to balance the final pool before sequencing. Library pools were sized to ~260 bp on a Pippin Prep instrument using 2% Pippin agarose gel. The sized libraries were evaluated on an Agilent High Sensitivity chip, quantified using real-time PCR, and used to prepare beads using a One-Touch 2 device. Beads were sequenced on an Ion Torrent Proton P1 chip. On average, 67 million reads were obtained per strain.

### Read Mapping and Gene Expression Quantification

RNA-seq reads were quality-trimmed using Trim Galore (31) and mapped to the mm10 reference genome or to the IAV PR8M genome using STAR (32). Counts were summarized at the gene level using the R-package *Rsubread* (33), normalized and log transformed using the R-package *DESeq2* (34), and batch-corrected using the *ComBat* function of the R-package *sva* (35, 36). For annotations of genes, ENTREZID from *Rsubread* were matched to RefSeq annotations using R-package *biomart* (37).

### Heritability Estimates for *Ace2*

Heritability of *Ace2* gene expression was estimated with GEMMA (38) using the following formula. This approach estimates the proportion of variance that can be explained (PVE) by genotypes covering nearly the whole genome:

$$\text{PVE}(\beta ,\text{u},\tau ):=\frac{\text{V}(\text{X}\beta +\text{u})}{\text{V}(\text{X}\beta +\text{u})+\tau^{-1}}$$

where “Xβ” is the sparse effects, “u” is the random effects, and “τ^−1”^ is the variance of the residual errors. GEMMA uses a Markov chain Monte Carlo method to estimate β, and u. We used the BXD genotype file generated on January 2017 (www.genenetwork.org/webqtl/main.py?FormID=sharinginfo&GN_AccessionId=600) and *Ace2* gene expression values across the 41 BXD family as input.

### Correlation Analysis

The correlation analysis in this study, including gene-gene and gene-phenotype rely on Pearson correlation coefficients (39). We reviewed data to ensure that outliers and distributional anomalies did not compromise downstream analyses.

### Weighted Gene Co-Expression Network Analysis

Weighted Gene Co-Expression Network Analysis (WGCNA) is a method that defines modules of co-expression, and that is often used to explore the associations between variation in mRNA levels and phenotypes of interest. It can be used to define hub genes and clusters in molecular and genetic networks. In this study, gene co-expression networks were constructed using the *WGCNA package* (40) following standard methods (https://horvath.genetics.ucla.edu/html/CoexpressionNetwork/Rpackages/WGCNA/Tutorials/). Modules were generated using the subset of 5500 H1N1-infected lung mRNAs that covaried well with *Ace2* expression (Pearson *r*) at a nominal *p* < 0.05. We used the standard WGCNA beta weighting parameter of 8 to achieve close to a scale-free distribution.

### Gene Set Enrichment Analysis

To understand the biological function and clusters detected as *Ace2* covariates and WGCNA modules, we compared set membership to Kyoto Encyclopedia of Genes and Genomes (KEGG) pathways and to the mammalian phenotype ontology (MPO) using WebGestalt (http://www.webgestalt.org) (41). This analysis uses a hypergeometric statistic test to generate adjusted *p* values and enrichment ratios. In this analysis, we used only protein coding genes as the reference background and a threshold minimum of five genes/transcripts per category.

### Quantitative Trait Loci Mapping

Quantitative Trait Loci (QTLs) define chromosomal regions that contribute to genetic variation in levels of gene expression, endophenotypes, and higher order outcome variables such as body weight loss and survival after infection. QTL mapping exploited methods in our GeneNetwork (www.genenetwork.org) (42, 43). Linkage statistics were computed initially using Haley-Knott equations (44), and genome-wide significance levels were estimated from 2,000 permutations of trait values. We used the new BXD genotype file of January 2017 (22). Those traits associated with significant and suggestive QTLs or expression QTLs (eQTLs) using the Haley and Knott regression were validated using GEMMA (38). GEMMA uses a linear mixed model mapping algorithm that accounts for complex kinship among strain genometypes.

### Genetic Variations Between B6 and D2

Sequence differences segregating among BXDs are taken from our previous work (45). Here, we focus exclusively on protein-coding variants—nonsense, missense, and frameshift mutations.

### Bayesian Network Construction

The Bayesian Network Webserver (BNW, compbio.uthsc.edu/BNW) (46–48) was used to create and evaluate plausible causal models among genes, transcripts, and outcome measures. The 100 highest scoring networks were used for model averaging, and those directed edges—unidirectional connections between nodes—with posterior probabilities greater than 0.8 were retained in the final model. We constrained each node to have at most four direct parents. We assigned nodes into one of three tiers of the Bayesian network: 1) SNP variant within selected QTL, 2) mRNA expression traits in a molecular endophenotype tier, and 3) the disease outcome measures—*Body Weight Loss* in the third tier. We allowed causal connections going from the first to second, and from second to the third tiers.

### RNA-Seq Time-Course Data for H1N1 Infection of the Parental Strains

Lung RNA-seq data was generated previously in our laboratory (20). In brief, the two parental strains, B6 and D2, were infected with the H1N1 virus at 10–12 weeks of age as described above. RNA was isolated from lungs of B6 at 1, 3, 5, 8, and 14 dpi and from lungs of D2 at 1, 3, and 5 dpi. Three RNA samples per strain and per time point were used for library preparation and sequencing. Raw counts were extracted, normalized, and log2 transformed with *DEseq2* (34) after adding 1 to all raw counts. In this study, we extracted the *Ace2* expression levels from this dataset. We also performed differential expression analysis of mice at 3 dpi and their mock treated controls using *DESeq2* (34) using strain as a covariate. The resulting *p* values were adjusted using the Benjamini and Hochberg’s approach (49) and are referred throughout this paper as adj. *p* values. We reported transcripts with adj*. p* < 0.05, and fold change (FC) > 1.3 as differentially expressed genes (DEGs). This data set was also used for evaluating the gene expression patterns post-infection.

### Transcriptome Data for SARS-CoV Infected Primary Human Airway Epithelial (HAE) Cells

The experimental details for this study are described in ref. (50). In brief, human tracheobronchial epithelial cells were resected from airway specimens of patients undergoing surgery. Primary cells were expanded to generate passage-1 cells, and passage-2 cells were plated at a density of 2.5 × 10^5^ cells per well. Human airway epithelium (HAE) cultures were generated by provision of an air-liquid interface for 6 to 8 weeks to form well-differentiated, polarized cultures. Wild-type infectious clone-derived SARS-CoV (icSARS-CoV) and ic-SARS-CoV ORF6 were derived from infectious clone constructs as described (50). Bat-SARS-CoV is comprised of the HKU3 backbone and SARS-CoV receptor binding domain and was derived from an infectious clone as described in ref. (51). Triplicate cultures were infected with a multiplicity of infection 3 (MOI 3) icSARS-CoV, and icSARS-CoV ORF6 on the apical surface. Total RNA was isolated from infected cells, and equivalent amounts of RNA from three biological replicates from each condition were pooled. Transcriptome profiling was performed using Agilent 4x44K whole human gene expression arrays. Raw data were downloaded from GEO (GSE47963_series_matrix.txt.gz). Expression values were normalized and sample descriptions quality controlled. A strong batch effect with respect to the donor from which HAE were derived was corrected using the R package *sva* (35, 36). Rows with no gene description were deleted. DEGs were determined using the R package *Limma* (52).

### Data Availability

The RNA-seq data from infected BXD strains has been deposited at GeneNetwork.org under the accession ID GN807. The summarized RNA-seq data as entered in GN can be accessed and downloaded at www.genenetwork.org/webqtl/main.py?FormID=sharinginfo&GN_AccessionId=807&InfoPageName=HZI_LungBXD_RNA-Seq_1116.

RNA-seq data for infected B6 and D2 parents are available at NCBI GEO under the accession number GSE66040. Array data from infected primary HAE cells are available at GEO under accession number GSE47963.

## Results

### *Ace2* Expression Level Decreased in Both B6 and D2 After H1N1 Infection

**Figure 1:**
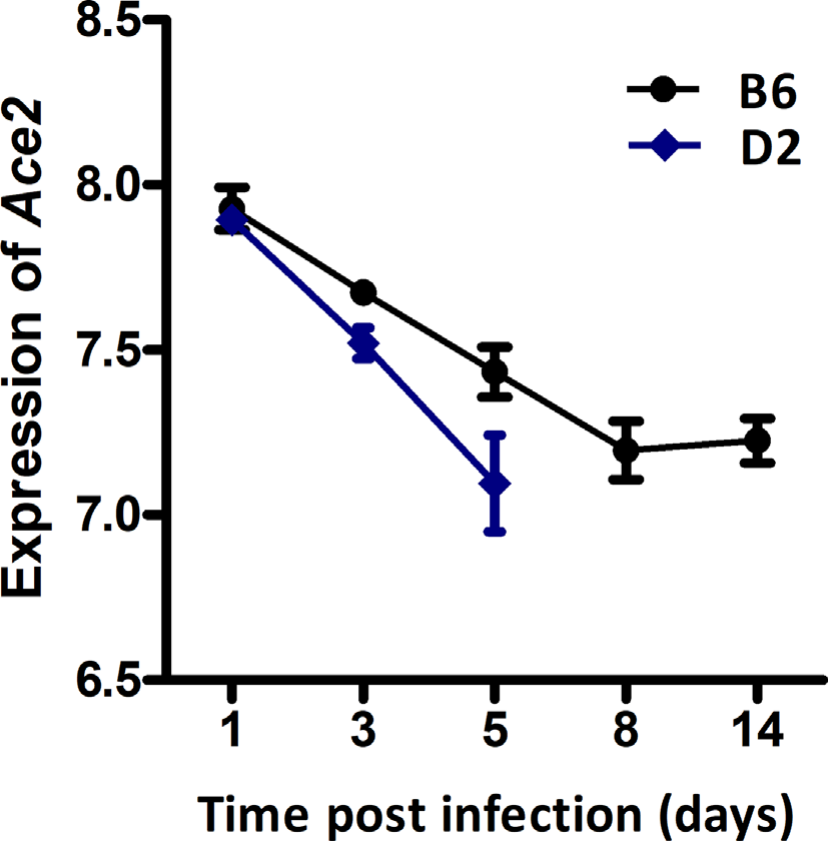
Line charts of the *Ace2* expression level in strains C57BL/6J (B6) and DBA/2J (D2) after H1N1 infection. Lung transcriptomes were quantified with RNA-seq for B6 and D2 strains at 1, 3, 5, 8, and 14 dpi of H1N1 infection. Three female mice (8–12 weeks of age) per strain were used per time point. The x-axis shows the time point (days) post the infection. The *y*-axis shows the expression level of *Ace2* on a log2 transformed scale.

We have previously generated H1N1-inflected lung RNA-seq for B6 and D2 parental strains at 1, 3, 5, 8, and 14 dpi. The B6 maternal parent of the BXDs is resistant to H1N1 infection, whereas the D2 paternal parent is highly susceptible to low virulent PR8 (H1N1) virus. Here, we have exploited these parental time-series data to define the *Ace2* mRNA post-infection dynamic response. Overall, expression of *Ace2* in lungs dropped progressively in both strains after infection (**Figure 1**). In B6, *Ace2* transcript reached its nadir at 8 dpi (7.20 ± 0.15 SD log2 units) and stayed low at 14 dpi (7.23 ± 0.12). Expression relative to 1 dpi was significantly lower at the following time points: 3 dpi (*p* = 0.026, log2FC = −0.25), 5 dpi (*p* = 0.008, log2FC = −0.49), 8 dpi (*p* = 0.003, log2FC = −0.73), and 14 dpi (*p* = 0.002, log2FC = −0.70). In comparison, the same infection titer was lethal for D2 which died at 5 dpi, and this was matched with lower expression of *Ace2* (7.10 ± 0.25) at 1 dpi. Statistically, *Ace2* expression 3 dpi (*p* = 0.003, log2FC = −0.37) and 5 dpi (*p* = 0.006, log2FC = −0.80) were significant reduced compared to 1 dpi. Although the differences in *Ace2* expression between B6 and D2 at 3 dpi and 5 dpi were not statistically significant, D2 mice had a consistently more rapid decline in expression compared to B6.

### *Ace2* Expression Exhibited Variability Across BXD Strains at 3 dpi

**Figure 2:**
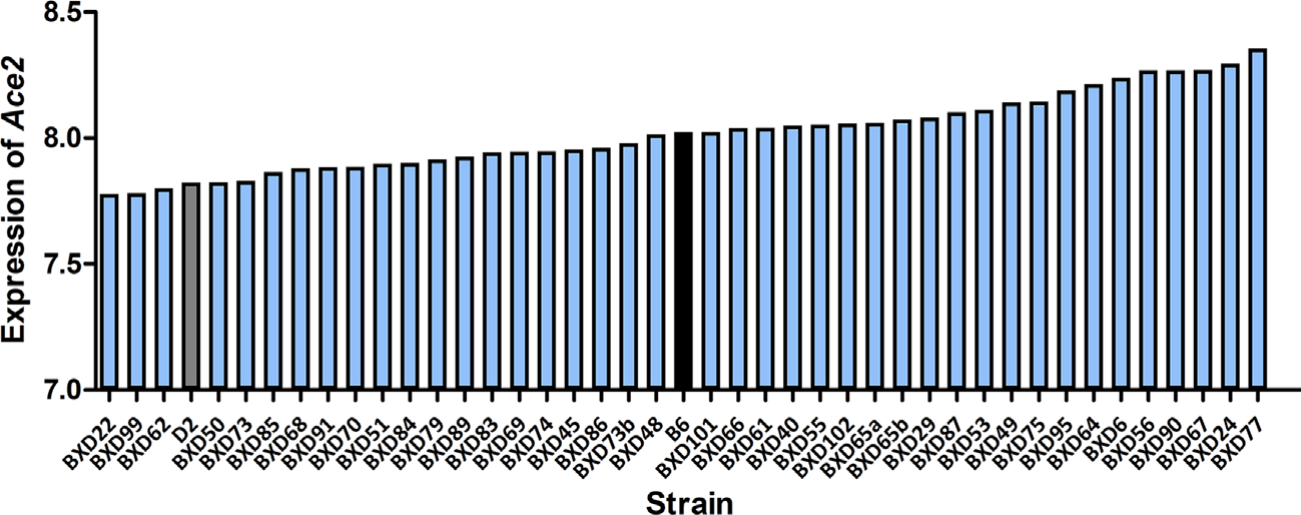
Expression of *Ace2* in the BXD family. Bar plots of the pulmonary *Ace2* expression levels across the BXD family at 3 dpi. The x-axis shows BXDs strains and the two parental strains. The y-axis shows the normalized log2 expression levels of *Ace2*.

All infected D2 mice died at 5 dpi. We therefore generated RNA-seq data at 3 dpi for infected BXD strains. Changes in *Ace2* transcript level after infection varies considerably across the BXD family with a 1.5-fold range (**Figure 2**). Mean expression of *Ace2* across all 43 strains is 8.02 ± 0.15 SD. BXD22 has the lowest expression of 7.78 and BXD77 has the highest expression of 8.36. The estimated heritability of *Ace2* expression variation is ~0.18, suggesting that expression of *Ace2* is partly impacted by genetic factors.

### *Ace2* Is Associated With Disease Severity After Infection

**Figure 3:**
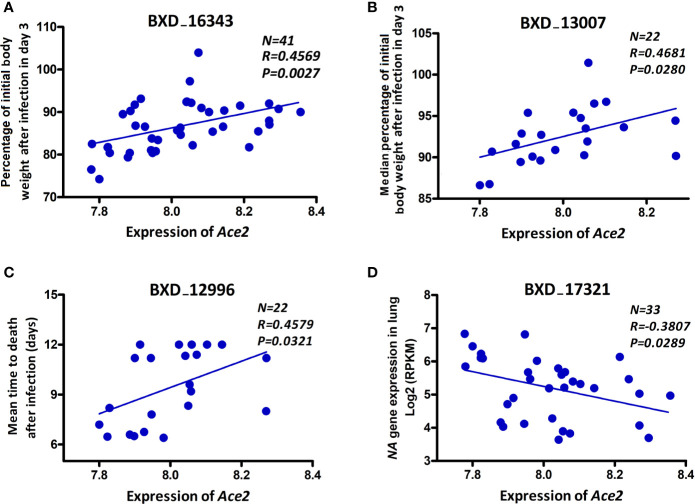
*Ace2* is associated with disease severity after infection. Scatter plots of the correlations of *Ace2* expression with body weight loss **(A)**, median body weight loss **(B)** at 3 dpi, mean time to death **(C)**, and viral neuraminidase (*NA)* gene expression levels **(D)**. Pearson correlation coefficient was used to determine the relationship. Gene expression levels represent normalized log2 values. GeneNetwork BXD phenotype identifiers (e.g., ID “BXD_16343”) are at the top of each plot. Number of strains (N), Pearson correlation R-value (R), and Pearson correlation *P*-value (P) are indicated. BXD trait 16343 and BXD trait 17321: one mouse per strain, BXD trait 13007 and BXD trait 12996: five mice per strain.

In order to investigate whether there is an association of *Ace2* expression with severity of disease and/or viral replication as infection progresses, we computed correlations of *Ace2* expression against body weight loss, mean time to death, and viral gene expression levels (as a surrogate for viral load). *Ace2* expression was significantly correlated with both body weight loss (*r* = 0.575, *p* = 0.008, **Figure 3A**) and median body weight loss (*r* = 0.468, *p* = 0.028, **Figure 3B**) at 3 dpi. Further, *Ace2* was positively and significantly correlated with the mean time to death (*r* = 0.458, *p* = 0.032, **Figure 3C**). In contrast, *Ace2* had a negative significant correlation with virus NA gene expression levels (*r* = -0.381, *p* = 0.029, **Figure 3D**).

### *Ace2*-Correlated Transcripts Are Significantly Involved in Immune System-Related Networks

**Figure 4:**
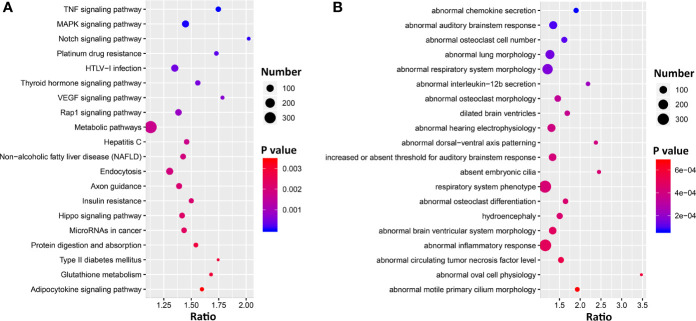
Bubble charts of the top 20 KEGG pathways **(A)** and 20 Mammalian Phenotype Ontologies **(B)** enriched for *Ace2* significantly correlated genes. Gene over-representation analysis for KEGG pathway and Mammalian Phenotype Ontology of the *Ace2* correlated transcripts (*p* < 0.05) were performed with WebGestalt (www.webgestalt.org). The *x*-axis represents the enrich ratio and the *y*-axis represents enriched pathways/terms. The size of the dots denotes the number of genes and the color denotes the *p*-value.

To gain insight into the pulmonary networks and biological functions that *Ace2* is involved in after viral infection, we computed Pearson correlation coefficients against the entire lung transcriptome. This analysis returned 5,500 transcripts nominally correlated with *Ace2* (*p* < 0.05, **Supplemental Data 1**). A total of 5,118 transcripts with Entrez gene IDs were submitted to WebGestalt (http://www.webgestalt.org/) for gene ontology analyses. The analysis highlighted strong enrichment in TNF signaling, MAPK signaling, Notch signaling pathways, HTLV-I infection, and VEGF signaling pathway (**Figure 4A**). Furthermore, we also found that these genes were enriched for categories related to chemokine secretion, lung morphology, and respiratory system morphology (**Figure 4B**).

Forty-eight genes are within the TNF signaling pathway (**Supplemental Data 1**), of which most have transcripts that covary negatively (*n* = 35) with *Ace2* (*p* < 0.05), including tumor necrosis factor (*Tnf, r* = −0.414, *p* = 0.005). Ninty-two genes are part of the extended MAPK signaling network (**Supplemental Data 1**). Of these *Tgfb1* (*r* = −0.459, *p* = 0.002) and 59 other transcripts covary negatively with *Ace2*. Finally, 25 genes are part of Notch signaling pathways (**Supplemental Data 1**), and again the majority of transcripts (*n* = 21) covary negatively with *Ace2*.

### Co-Expression Network Analysis Narrowed Down *Ace2*-Correlated Transcripts

**Figure 5:**
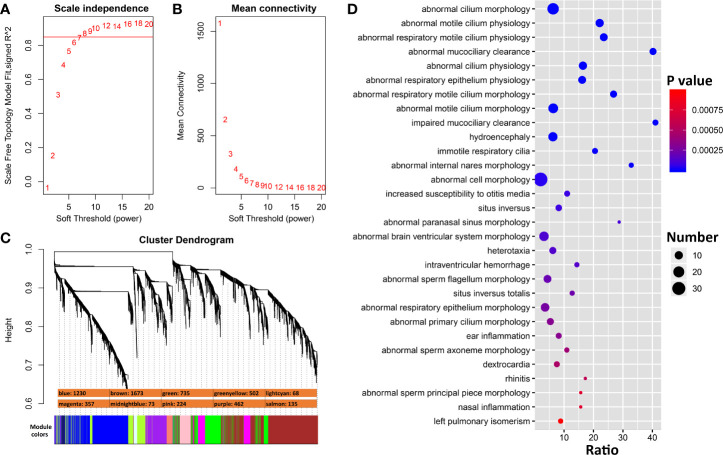
WGCNA modules associated with *Ace2* expression in H1N1 infected lung. **(A)** The soft thresholding index R^2^ (*y*-axis) as a function of different powers β (x-axis) indicating the threshold of 8 chosen. **(B)** Mean connectivity (degree, y-axis) as a function of the soft-thresholding power (*x*-axis). **(C)** 10 co-expression modules identified from *Ace2* correlated genes. Modules are color coded as shown. The number of genes in each module is also indicated. **(D)** Bubble charts of top 30 enriched Mammalian Phenotype Ontologies of the purple module. Analysis by WebGestalt.

To identify genes that interact more directly with *Ace2*, we constructed co-expression modules using WGCNA with *Ace2* correlated transcripts. The top most variant 5,500 transcripts were parsed into 10 co-expression modules using a soft-thresholding power of 8 (**Figures 5A–C**). Modules have a wide range of sizes, with a *brown* module containing 1,673 transcripts and *lightcyan* containing only 68 transcripts. *Ace2* was located in the *purple* module with 461 other genes/transcripts (**Figure 5C**). Genes in this module are significantly involved in cilium function (**Figure 5D**)

**Figure 6:**
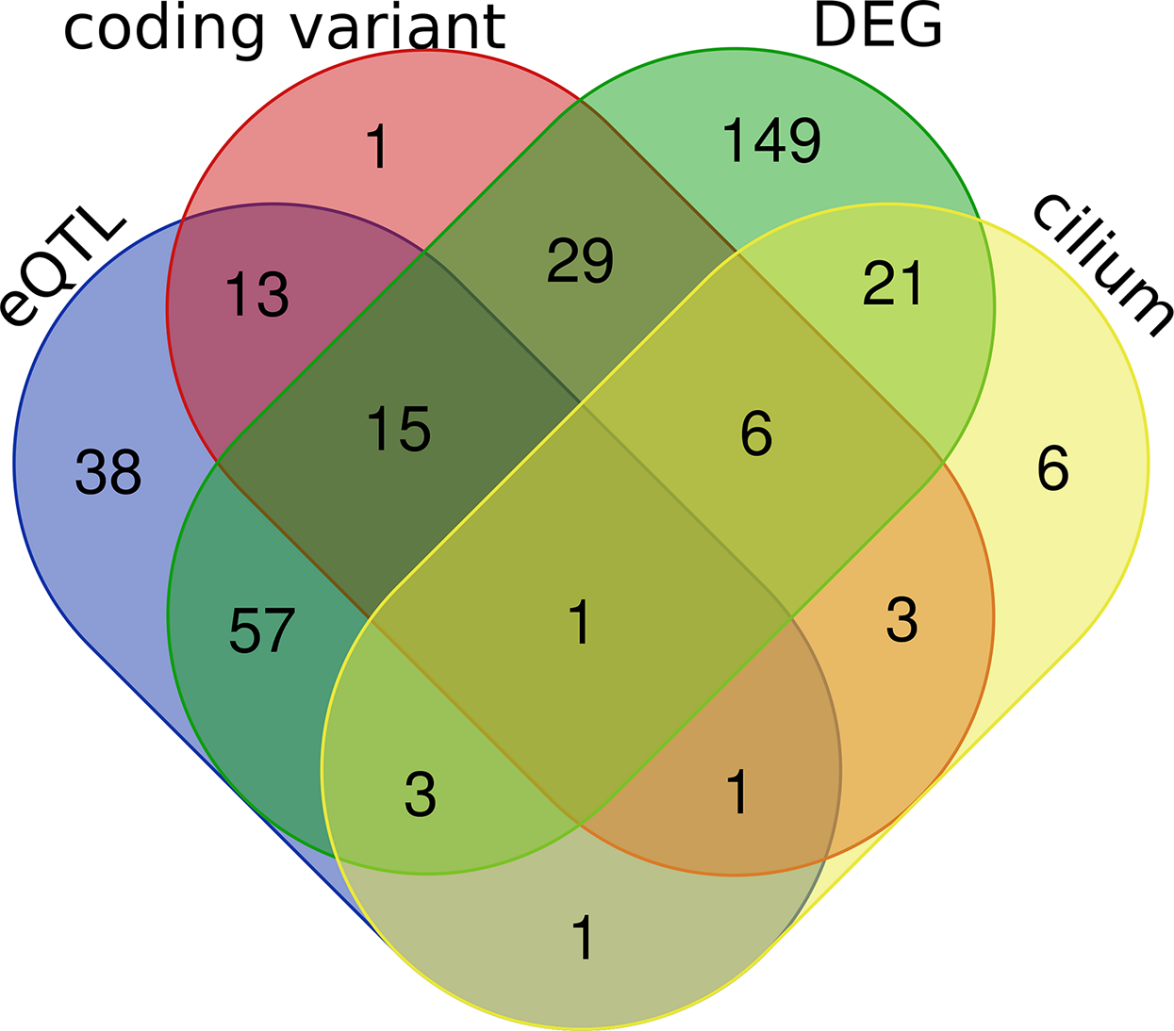
Venn diagrams of the purple module genes. A total of 462 genes were included in the purple module. Four criteria were applied to explore the module genes, including coding sequence variants between parental strain B6 and D2 (coding variant), eQTL (LRS > 15) regulation in H1N1 affected lung tissue (eQTL), cilium functional relevance (cilium), and differential expression (adj. *p* < 0.05 and FC > 1.3) between infected and normal controls in parental strains (DEG). The Venn diagram shows the number of overlapping genes among the four categories.

In subsequent analyses we focused on transcripts within the *purple* module (**Supplemental Data 2**). We carried out differential expression analysis between cases and mock-infected controls and defined 281 transcripts (61% of the module) with differential expression (adj. *p* < 0.05 and FC > 1.3). eQTL mapping of the H1N1-infected whole lung transcriptome defined 23 cis-modulate transcripts and 106 *trans*-modulated transcripts, of which 69 are likely to harbor coding sequence variants. A subset of 42 of these genes has been implicated in cilium function. By comparing the above four categories (**Figure 6**), 31 cilium genes were differentially expressed (**Table 1**). Of these, *Sox11*, *Tff2*, *Rnf128*, and *Muc5b* are either *cis*- or *trans-*controlled, whereas *Sox11*, *Tmprss2*, *Ulk4*, *Cckar*, *Wdpcp*, *Ift140*, and *Ccdc40* contain functional variants (**Table 1**).

**Table 1:** Lists of prioritized genes in the purple module that were differentially expressed (adj. *p* < 0.05 and FC > 1.3) and implicated in cilium biological functions.

Gene ID	Symbol	Description	Log2 FC	Adj.p	eQTL	Functional Variant
20666	*Sox11*	SRY-box 11	−1.19	1.95E-12	cis	Nonsynonymous
21785	*Tff2*	trefoil factor 2 (spasmolytic protein 1)	−1.01	5.38E-04	trans	–
66889	*Rnf128*	ring finger protein 128	−0.77	2.81E-05	trans	–
74180	*Muc5b*	mucin 5, subtype B, tracheobronchial	0.54	1.13E-02	trans	–
50528	*Tmprss2*	transmembrane protease, serine 2	−1.06	8.58E-10	–	Nonsynonymous
209012	*Ulk4*	unc-51-like kinase 4 (C. elegans)	−0.87	3.43E-07	–	Nonsynonymous
12425	*Cckar*	cholecystokinin A receptor	−0.73	1.91E-05	–	Nonsynonymous
216560	*Wdpcp*	WD repeat containing planar cell polarity effector	−0.69	2.36E-06	–	Nonsynonymous
106633	*Ift140*	intraflagellar transport 140 homolog (Chlamydomonas)	−0.68	3.50E-07	–	Nonsynonymous
207607	*Ccdc40*	coiled-coil domain containing 40	−0.66	1.06E-03	–	Nonsynonymous
320752	*Dpy19l2*	dpy-19-like 2 (C. elegans)	−1.81	2.13E-03	–	–
51938	*Ccdc39*	coiled-coil domain containing 39	−1.30	1.15E-13	–	–
244653	*Hydin*	hydrocephalus inducing	−1.30	8.51E-16	–	–
74362	*Spag17*	sperm associated antigen 17	−1.29	9.27E-13	–	–
73873	*Fam161a*	family with sequence similarity 161, member A	−1.28	1.70E-09	–	–
320662	*Casc1*	cancer susceptibility candidate 1	−1.27	2.44E-08	–	–
66722	*Spag16*	sperm associated antigen 16	−1.27	1.61E-10	–	–
74934	*Armc4*	armadillo repeat containing 4	−1.21	8.50E-09	–	–
18703	*Pigr*	polymeric immunoglobulin receptor	1.18	1.10E-09	–	–
69707	*Iqcg*	IQ motif containing G	−1.18	2.97E-20	–	–
78801	*Ak7*	adenylate kinase 7	−1.17	4.75E-09	–	–
27078	*B9d1*	B9 protein domain 1	1.09	1.00E-02	–	–
68922	*Dnaic1*	dynein, axonemal, intermediate chain 1	−1.07	3.67E-08	–	–
226265	*Eno4*	enolase 4	−0.98	1.49E-09	–	–
71877	*Efhc1*	EF-hand domain (C-terminal) containing 1	−0.92	3.77E-11	–	–
75533	*Nme5*	expressed in non-metastatic cells 5	−0.82	5.92E-05	–	–
75050	*Kif27*	kinesin family member 27	−0.71	3.05E-03	–	–
70008	*Ace2*	angiotensin I converting enzyme (peptidyl-dipeptidase A) 2	−0.61	9.88E-05	–	–
20878	*Aurka*	serine/threonine kinase 6	0.60	2.36E-02	–	–
22062	*Trp73*	transformation related protein 73	−0.60	1.81E-03	–	–
252973	*Grhl2*	grainyhead-like 2 (Drosophila)	−0.44	5.36E-04	–	–

### The Differentially Expressed Cilium Function Related Genes in the *Purple* Module Were Replicated in SARS-CoV-Infected HAE Cells

**Figure 7:**
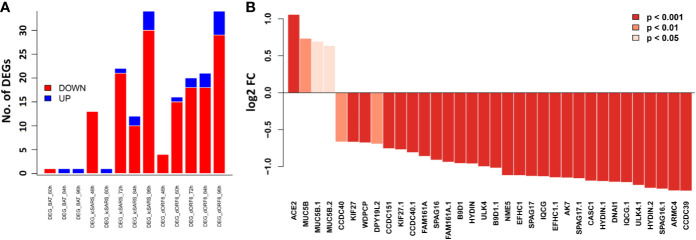
Bar plots of the cilium function related genes in the SARS-CoV infected HAE cells. Primary human HAE cells were infected with MOI 3 SARS-CoV (icSARS-CoV), ic-SARS-CoV ORF6 and BAT-SARS_CoV and gene expression was analyzed by arrays. **(A)** Number of up-and downregulated differentially expressed genes for cilium function related genes that had orthologous genes in humans and were represented on the array (adj. *p* < 0.05 and log2 FC > 0.58); y-axis: number of differentially expressed genes. **(B)** Log2 FC differences in expression values between HAE cells infected with icSARS-CoV at 96_h pi and mock-treated controls for cilium function related genes that had orthologous genes in humans and were represented on the array; y-axis: differences in log2 FC expression levels between groups. Adjusted p-values were taken from the Limma analysis of the entire transcriptome data.

In order to evaluate whether cilium related genes (**Table 1**) are also modulated in humans, especially in the context of SARS-CoV infection, we analyzed our transcriptome data set for HAE cells infected with SARS-CoV (icSARS-CoV), ic-SARS-CoV ORF6 and BAT-SARS_CoV (50). Very few of these transcripts were significantly affected after infection with BAT-SARS (**Figure 7A**). However, most of them were differentially expressed in icSARS-CoV and ic-SARS-CoV ORF6-infected HAE cells when compared to mock control, with 21 transcripts modulated at 96 h after infection (**Figure 7A**). More importantly, those overlapping transcripts, except *Ace2*, exhibited the same up- or down-expression patterns in both HAE cells and our mouse model (**Table 1** and **Figure 7B**). *ACE2* was up-regulated in HAE cultures whereas in whole mouse lungs it was down-regulated. This is an important observation because HAE cultures consist almost exclusively of airway epithelial cells, with few if any interstitial cells, endothelial cells, resident and infiltrating immune cells. This indicates that *ACE2* networks detected in whole lungs are much more complex than those detected in isolated epithelial cells. It may be the case that *ACE2* is downregulated in all cells in lung after infection, but alternatively, *ACE2* may be up-regulated only in infected cells (50, 53). In the lungs, infected cells represent a minority of all cells. Since our lung transcriptome quantifies steady-state expression changes of all cell types, it is conceivable that we do not detect up-regulation in a smaller population of infected cells. Besides, two recent studies (54, 55) show alternative splicing of ACE2 in humans, up-regulated by both viruses and IFNs. Therefore, it may be important to quantify different *Ace2* isoforms’ expression in infected mouse lungs by immunohistochemistry, by single-cell methods, or by *in situ* hybridization to resolve specificity and kinetics of responses.

### Bayesian Network Analysis of Causal Pathways Underlying Post-Infection Weight Loss

**Figure 8:**
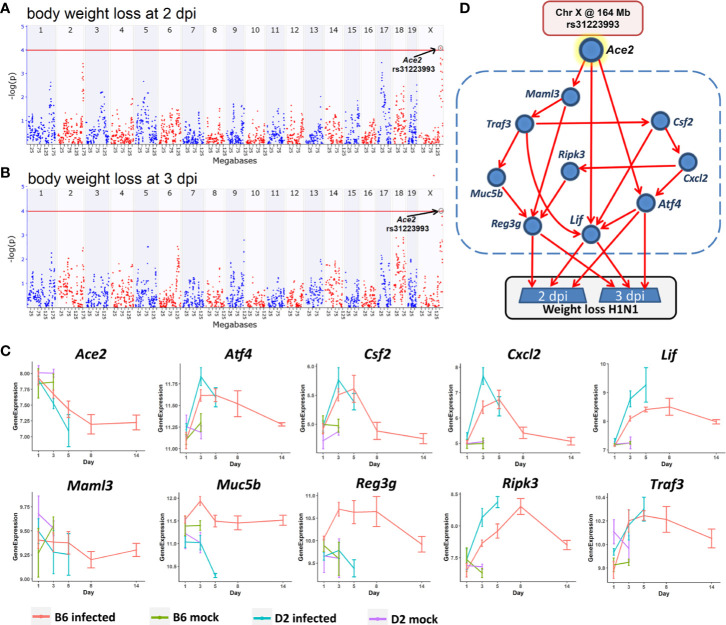
A Bayesian network for the influenza disease severity in the BXD family. QTL maps of body weight loss at 2 **(A)** and 3 dpi **(B)** with peaks on Chr X at 164 Mb, respectively. The *x*-axis is chromosome position on a megabases (Mb) scale, while the *y-*axis gives the –log(p) of linkage. Each point on this plot is a single SNP marker used to evaluate linkage. The red line indicates the genome-wide significance threshold of ~4. **(C)** Kinetics of expression changes of the ten transcripts that are part of the Bayesian network. The *x*-axis defines days after infection, and the *y*-axis defines expression on a log2 scale. **(D)** Candidate Bayesian network comprising a top tier of genotypes on Chr X that predict weight loss (rs31223993), a middle tier within the blue dashed box of gene expression endophenotypes, and a bottom tier of body weight loss.

Body weight loss is an important indicator of post-infection severity. We performed QTL mapping for body weight changes after infection. This allowed us to identify locus on Chr X at 164 Mb that clearly modulate body weight loss at 2 and 3 dpi (**Figures 8A, B**), respectively. This locus is precisely aligned to the chromosomal position of the *Ace2* gene (Chr X at 164.2 Mb), which supports a central hypothesis of this study—that variants in or near *Ace2* influence influenza disease severity.

In order to investigate how this locus modulates influenza disease severity, hereafter referred to as body weight loss, we created a Bayesian network model (**Figure 8D**) comprising genotypes at this QTL location (Chr X: rs31223993) plus its candidate gene *Ace2* as a genetic driver, and body weight loss as the key outcome response. We tested causal networks that included nine transcripts as putative disease mediators. All nine are highly expressed in the lung, have a significant correlation with *Ace2* and body weight loss at 2 and 3 dpi (*p* < 0.05), and have a significant expression change (FC > 1.3 and adj. *p* < 0.05) after infection at multiple time points between the infected and the mock controls (**Figure 8C**). Moreover, all of these transcripts and their cognate genes have been implicated in functions related to the infection related pathways (TNF signaling pathway, IL-17 signaling pathway, Th1 and Th2 cell differentiation).

The Bayesian network model (**Figure 8D**) linked the Chr X QTL for body weight loss outcome at 2 and 3 dpi with multiple gene mediators, including C*sf2, Cxcl2*, *Maml3*, *Muc5b*, Ripk3, and*Traf3*. It is worth noting that any pathway leading from the major Chr X QTL to body weight loss must pass through *Atf4*, *Lif*, or *Reg3g.* For instance, one causal path for body weight loss begins at the Chr X QTL genotype (rs31223993), continues to the expression of *Ace2* and *Lif*, and finally impacts phenotype outcomes.

## Discussion

Gene variants significantly influence the susceptibility of humans to influenza virus infections (56). This is just as true in mouse and other organisms (19). In our case, it is especially true of the two parents of the BXD family (20). Here, we have systemically analyzed the potential causal role of *Ace2* expression in disease sensitivity and severity. Our focus is on networks of transcripts that covary with differences in *Ace2* expression after H1N1 infection. Expression of *Ace2* in lung is markedly but differentially decreased in a time-dependent manner. Expression variation is negatively correlated with body weight loss and with differences in survival time after infection. More importantly, the locus that modulates body weight loss at 2 and 3 dpi is co-located precisely with the location of *Ace2* on distal Chr X. These results strongly suggest that *Ace2* polymorphisms, or perhaps different isoforms as in humans (54), are linked to H1N1 disease progression, and that lower levels of *Ace2* may play a role in virus-induced ALI. This idea is corroborated by studies of *Ace2* ^−/−^ knockouts infected with influenza H7N9 (17) or with respiratory syncytial virus (16). In both cases, lung pathology is more severe in the knockouts. These observations suggest that *Ace2* may mediate virus-induced ALI (16, 17). However, the underlying processes remain elusive and direct causes, versus reactive changes, are still not proved.

### *Ace2* Correlated Genes Are Implicated in Inflammatory Related Pathways

By using 41 isogenic RI strains, we defined *Ace2*-associated modules and molecular networks. We identified a total of ~5,000 *Ace2*-correlated transcripts in infected lungs. As a set they are enriched in genes associated with respiratory and inflammatory networks and in several well understood pathways, including TNF, MAPK, and Notch signaling pathways. Extending this type of analysis to the corresponding proteomic, metabolomic, and metagenomic levels would provide a complementary and more accurate understanding of lung pathogenesis.

Key factors associated with H1N1-virus-induced lethality include virus-induced inflammatory cytokine storm and subsequent tissue destruction. Tumor necrosis factor (*TNF*) is a cytokine with multiple effects, including promoting cell growth, differentiation, apoptosis, and inducing inflammation (57, 58). *TNF-α*, a member of the *TNF* family, can activate caspase protease, JNK, and transcription factor NF-κB, thereby amplifying its roles in cytotoxicity, antiviral action, and immuno-regulation. A previous study reported that inhibition of *Tnf-α* can protect mice from lethal H1N1 infection (59). In the present study, we found *Ace2* was negatively correlated (*r* = −0.414, *p* = 0.005) with the expression of *Tnf* and 34 other TNF signaling pathway genes, suggesting that *Ace2* may dampen excessive inflammatory immune responses by downregulating this signaling pathway and mitigating immune-mediated tissue destruction. We also highlighted 92 genes in the MAPK signaling pathway that covary with *Ace2*, including *Tgfb1* (*r* = −0.459, *p* = 0.002). Of note, it was shown that IAV can activate this pathway to support its replication (60). The authors proposed a model in which *Tgfb1* acts as a pro-viral host factor by suppressing early immune responses, creating an environment conducive for IAV infection (61). Moreover, another set of 25 *Ace2*-correlated transcripts involved in the Notch signaling, most with negative covariation. This pathway is a bridge between antigen-presenting cells (APCs) and T cell communication circuits and plays a critical role in driving the immune system to overcome infectious disease (62).

### WGCNA Identifies *Ace2* Co-Expressed Genes in the Respiratory Epithelium System

There are essentially two parallel perturbation in this experiment—the actual infection with H1N1, and the natural genetic variation among these 43 diverse strains and their genomes. When performing WGCNA, a core of 461 transcripts covaried with *Ace2* after infection was identified (*purple* module). This set of covarying transcripts contained 31 genes that were related to ciliary function, including *Ace2* itself. *Ace2* expression may also modulate respiratory epithelial activity, although we stress that the causal polarity is still unknown.

Among the 31 prioritized cilium function related genes from the purple module (**Table 1**), *Rnf128*, *Muc5b*, *Sox11*, and *Tff2* showed *cis*- or *trans-*regulation, and *Tmprss2*, *Ccdc40, Ulk4*, *Cckar*, *Wdpcp*, and *Ift140* contained functional variants. *Rnf128* encodes a ring-finger-containing protein which has been shown to possess E3 ubiquitin ligase activity (63). *RNF128* is implicated in the induction of T cell anergic phenotype through inhibiting activation-induced IL2 and IL4 cytokine production (64) and mediating T cell receptor-CD3 degradation (65). In a recent study, Song et al. (66) reported that *RNF128* promotes lysine-63-linked ubiquitination and activation of a critical kinase TBK1, thereby augmenting the induction of type I interferon antiviral cytokines that curb viral replication. The authors’ *in vivo* experiments further showed that *Rnf128* deficient mice were more susceptible to vesicular stomatitis virus (an RNA virus) and herpes simplex virus (a DNA virus). *MUC5B* (mucin 5, subtype B, tracheobronchial), encodes a member of the mucin family of proteins, and is associated with idiopathic pulmonary fibrosis in humans (67). Overexpression of this gene in mice causes mucociliary dysfunction, and enhances lung fibrosis (68). It was reported to be required for airway defense in another study (69). *TMPRSS2* (transmembrane protease, serine 2) encodes a protein that belongs to the serine protease family. *Tmprss2* knockout mice show reduced lung inflammation with IAVs (H1N1, H3N2, or H7N9) infection and exhibit decreased susceptibility to virus-induced morbidity/mortality (70). Studies also demonstrated that SARS coronavirus engages *ACE2* as the entry receptor on the cell surface (8, 9) and employs the *TMPRSS2* for Spike protein priming, which facilitates viral entry and infection (71, 72). *CCDC40* (coiled-coil domain containing 40) encodes a protein that is essential for motile cilia function. According to the OMIM database, this gene is associated with recurrent respiratory infections (HP:0002205) and abnormal axonemal organization of respiratory motile cilia (HP:0012258). Malfunction of *Ccdc40* in mice resulted in left pulmonary isomerism and lung situs inversus (73). In addition, *Ulk4*, *Sox11*, and *Tff2* are associated with abnormal respiratory motile cilium (74), pulmonary hypoplasia (75), and abnormal adaptive immunity (76), respectively.

### Genetic Causal Pathways for the Host Responses to Infection

The innate immune system plays a crucial role in the early recognition and subsequent triggering of proinflammatory responses. Our Bayesian network model suggests that this response may be modulated in part by TNF signaling pathway related genes (*Atf4*, *Csf2*, *Cxcl2*, *lif*, and *Traf3*), particularly *Atf4* and *Lif*, two mediators for body weight loss at 2 and 3 dpi*. Atf4* encodes a transcription factor that was originally identified as a widely expressed mammalian DNA binding protein that could bind a tax-responsive enhancer element in the LTR of HTLV-1. Coronavirus replication is structurally and functionally associated with the endoplasmic reticulum (ER). The over-expression of the spike protein of SARS-CoV, MHV, and HCoV-HKU1 has been shown to induce potent ER stress in cell culture (77–80). In response to ER stress, phosphorylated eIF2α induces the expression of *ATF4* (81). A previous study has demonstrated the protective effect of Ang 1–7 against Ang II induced ER stress and endothelial dysfunction (82). It indicates that the expression levels of *Ace2* and *Atf4* show a negative correlation, which is consistent with the results of our analysis. Furthermore, as an ER sensor, *ATF4* defends lungs via induction of heme oxygenase 1 and its dependent responsiveness to ER stress is significantly impaired in the elderly (83). *Lif* (leukemia inhibitory factor) encodes a pleiotropic cytokine with roles in several different systems (http://www.informatics.jax.org/marker/MGI:96787). A study has shown that LIF is expressed in the airspace of pneumonic lungs and the endogenous LIF facilitates tissue protection during pneumonia (84). In addition, The LIF-STAT3 axis has been identified as a key determinant of lung injury (84). The proactive role of *Lif* on lungs during infection was also demonstrated by another study (85). Therefore, Metcalfe proposed to use LIF to protect the lungs and reduce disease severity caused by SARS-CoV-2 (86).

Immune responses not only act to control and eliminate infections, but can inflict collateral damage of host lungs and other tissues. A diversity of molecules, metabolites, and cell types are involved including some of genes implicated in host defense against pathogens (*Atf4*, *Cxcl2*, *Lif*, *Muc5b*, *Reg3g*, *Ripk3*, and *Traf3*) in the Bayesian network. The key genetic driver of this damage in the BXD family is a locus on Chr X at 164 Mb, precisely the position of *Ace2*. This root causal path to body weight loss is mediated in part by variation in *Ace2* and *Reg3g expression* (regenerating islet-derived 3 γ). The protein encoded by this gene is an antimicrobial lectin with activity against Gram-positive bacteria. *Reg3g* is a critical STAT3 target in lung epithelium, and is also highly expressed in gastrointestinal epithelium (87). Our previous study revealed that *Reg3g* was significantly up-regulated in B6 but not in D2 after the H1N1 infection. *Reg3g* knockout mice on the B6 background showed a decrease in body weight at 4–6 dpi. (20). STAT3-mediated induction of the antimicrobial protein REG3G is required for host defense against methicillin-resistant *Staphylococcus aureus* pneumonia (87). A study has shown that IL22 and IL6 influence *Reg3g* expression in the gut and lungs, respectively (88). Gastrointestinal delivery of recombinant REG3G decreased lung inflammatory gene expression and protected *Il22* ^−/−^ mice from weight-loss during infection (88).

### Implications for COVID-19

The ongoing outbreak of the COVID-19 caused by SARS-CoV-2 poses a global public health threat (89–91). SARS-CoV-2 entry into host cells is partly dependent on *ACE2* (10), the receptor for SARS-CoV (8, 9). How ACE2 interacts with other proteins involved in the progression of SARS-CoV-2-induced respiratory diseases remains to be determined. The first report based on autopsy samples demonstrated that the pathological characteristics of COVID-19 are similar to those of SARS-CoV and MERS-CoV infected patients (92). We therefore focused most attention on 31 prioritized genes involved in cilium function (**Table 1**) that are co-expressed with *Ace2* after H1N1 infection in mouse and in SARS-CoV-infected human HAE cells. Twenty out of these 31 transcripts are consistently up- or down-regulated, suggesting these two viruses share common *Ace2* networks that participate in the development and severity of lung pathology. In addition, our Bayesian network model also suggests several genes could be targeted for lung protection during SARS-CoV-2 infection, such as *Atf4, Lif*, and *Reg3g*.

## Data Availability Statement

The datasets presented in this study can be found in online repositories. The names of the repository/repositories and accession number(s) can be found at https://www.genenetwork.org/(GN807), https://www.ncbi.nlm.nih.gov/(GSE66040), and https://www.ncbi.nlm.nih.gov/(GSE47963).

## Ethics Statement

The animal work described herein was approved by the Institutional Animal Care and Use Committee (IACUC) at the University of Tennessee Health Science Center (UTHSC).

## Author Contributions

LL, KS, RW, and RB conceived the study. SB and AS conducted the experiments. FX, KS, YC, and RW performed data analysis. LL, FX, KS, RW, and JG wrote the manuscript. FX, KS, and JG prepared the figures and tables. RW, KL, DA, and CJ edited the manuscript. All authors contributed to the article and approved the submitted version.

## Funding

This work was supported by an intra-mural grant from the Helmholtz-Association (Program Infection and Immunity), a start-up grant from the University of Tennessee Health Science Center, a NIH/NIAID Research Grant U19-AI100625 to KS, and NIH/NIAID Research Grants U19-AI100625, U01 AI149644, and U19 AI 109761 to RB. GeneNetwork is supported by R01 GM123489 and the UT Center for Integrative and Translational Genomics. Provision of cells by Scott H. Randell was supported by grants from the Cystic Fibrosis Foundation (BOUCHE19R0 and NIH (DK065988).

## Conflict of Interest

The authors declare that the research was conducted in the absence of any commercial or financial relationships that could be construed as a potential conflict of interest.
